# Editorial Comment to Early return to continence and potency with use of dehydrated human umbilical cord graft at the time of robot‐assisted radical prostatectomy: A case study and analysis of relevant literature

**DOI:** 10.1002/iju5.12275

**Published:** 2021-03-03

**Authors:** Francesco Chiancone

**Affiliations:** ^1^ Urology Department AORN A. Cardarelli Naples Italy

Nowadays, patients have higher expectations from mini‐invasive surgery. Continence, potency, biochemical recurrence‐free survival, no postoperative complications, and negative surgical margins (so‐called “pentafecta”)^1^ represent current patients' expectations. Pentafecta achievements are viable tools to report outcomes after robot‐assisted radical prostatectomy (RARP).

Incontinence and erectile dysfunction can have serious impact on quality of life.^2^ Despite remarkable technical advances and improvements in pelvic reconstructive techniques,^3^ the continence and potency rates reach 96.4% and 89.8%, respectively.^1^

Urinary incontinence is however influenced by preoperative characteristics (such as age, body mass index, baseline urinary function, bladder stability, and prostate volume) and experience of the surgeon.

Preoperative erectile function, surgeon's surgical technique and experience are the most important predictors of postoperative sexual function.

Interestingly, social distancing during the COVID‐19 (COronaVIrus Disease 19) pandemic also influenced early urinary continence and desire for sexual rehabilitation in patients undergoing RARP.^2^

Despite nerve‐sparing techniques, erectile dysfunction can be related to neuropraxia of the neurovascular bundles caused by thermal damage or excessive traction to the nerves. Neuropraxia induces an inflammatory response that can lead to damage of the cavernosal nerves.

Based on these observations, Krol *et al*.^4^ described the use of dehydrated human umbilical cord (UC) graft in a 67‐year‐old patient who underwent bilateral nerve‐sparing RARP. The UC allograft was placed over the neurovascular bundle before posterior muscle‐fascial reconstruction. At 4 weeks follow‐up, the patient reported minimal stress urinary incontinence (SUI) utilizing 1 pad/day without notable leakage and erection satisfactory for penetrative sexual intercourse without the use of oral phosphodiesterase type 5 inhibitors. At 6 months follow‐up, complete continence and full sexual potency were achieved.

UC allografts contain a lot of growth factors and cytokines that control inflammation, promote angiogenesis and tissue recovery. In a large case–control study of 200 patients (*n* = 100/group),^5^ continence recovery rates were significantly better for patients receiving UC when compared to control groups, with particular benefit for patients >60 years old and with a body mass index ≥30 kg/m^2^. Despite this, there are no previous reports in the literature assessing potency recovery in patients receiving UC during RARP.

Currently, dehydrated human amnion/chorion membrane (dHACM) is most widespread used as allograft during RARP. However, UC is twice as thick as dHACM grafts which yields improved handling characteristics for robotic techniques and the ability to be secured with sutures. Moreover, UC contains higher amounts of extracellular matrix proteins and UC allografts have highest sterility assurance level.^4^

Further investigations in larger well‐controlled trials are necessary to confirm these preliminary results.

## Conflict of interest

The author declares no conflict of interest.
